# New evidence about the “dark side” of social cohesion in promoting binge drinking among adolescents

**DOI:** 10.1371/journal.pone.0178652

**Published:** 2017-06-02

**Authors:** Juliana Gabrielle Martins, Haroldo Neves de Paiva, Paula Cristina Pelli Paiva, Raquel Conceição Ferreira, Isabela Almeida Pordeus, Patricia Maria Zarzar, Ichiro Kawachi

**Affiliations:** 1 Pediatric Dentistry and Orthodontics, School of Dentistry, Federal University of Minas Gerais, Belo Horizonte, Minas Gerais, Brazil; 2 Department of Dentistry, School of Dentistry, Federal University of Jequitinhonha and Mucuri Valleys, Diamantina, Minas Gerais, Brazil; 3 Social and Preventive Dentistry, School of Dentistry, Federal University of Minas Gerais, Belo Horizonte, Minas Gerais, Brazil; 4 Department of Social and Behavioral Sciences, Harvard School of Public Health and Medical School, Harvard, Boston, Massachusetts, United States of America; Oregon Health and Science University, UNITED STATES

## Abstract

Adolescence is characterized by heightened susceptibility to peer influence, which makes adolescents vulnerable to initiating or maintaining risky habits such as heavy drinking. The aim of the study was to investigate the association of social capital with longitudinal changes in the frequency of binge drinking among adolescents at public and private high schools in the city of Diamantina, Brazil. This longitudinal study used two waves of data collected when the adolescents were 12 and 13 years old. At the baseline assessment in 2013 a classroom survey was carried out with a representative sample of 588 students. In 2014, a follow-up survey was carried out with the same adolescents when they were aged 13 years. The Alcohol Use Disorder Identification Test-C (AUDIT C) was employed for the evaluation of alcohol intake. Our predictor variables included sociodemographic and economic characteristics (gender, type of school, mother's education, family income) and Social Capital. For evaluation of social capital, we used the Social Capital Questionnaire for Adolescent Students (SCQ-AS). Descriptive and bivariate analyzes were performed (p <0.05). The log-binomial model was used to calculate prevalence ratios (PR) and 95% confidence intervals. The two-tailed *p* value was set at <0.05. The prevalence of binge drinking in 2013 was 23.1% and in 2014 the prevalence had risen to 30.1%. Gender (PR 1.48; 95% CI 0.87–2.52) and socioeconomic status (type of school and mother’s education) were not associated with the increase in the frequency of binge drinking. However, higher social capital was significantly associated with an *increase* in binge drinking by students. Adolescents who reported that they had an increase in social cohesion in the community/neighborhood subscale were 3.4 times more likely (95%CI 1.96–6.10) to binge drink themselves. Our results provide new evidence about the “dark side” of social cohesion in promoting binge drinking among adolescents.

## Introduction

Adolescence, more than in any other developmental stage, is characterized by heightened susceptibility to peer influence [1], which makes adolescents vulnerable to initiating or maintaining risky habits such as heavy drinking [2]. People are likely to engage in behaviors that match their perceptions of what is “normative,” especially characteristics of those who represent idealized identities, such as high-status peers. Many deviant and risky behaviors are associated with high peer status and it is suggested that some adolescents strive to imitate their high status peers through a process of social comparison [3] which means that adolescents contrast their own sense of values, interests, beliefs, and behaviors with their perceptions of others and, in consequence of this, construct a sense of identity.

Various risk factors for problem drinking among youth have been identified by researchers. The emphasis across studies has been on risk and protective factors [4,5]. There is increasing evidence that social environmental factors influence alcohol consumption and harms among youth. Social capital is one contextual factor that has been related to binge drinking—defined as consuming 5 or more drinks on one occasion—[6] among adolescents. Social capital is defined as the resources—such as social support, trust, and information channels—accessed by individuals through their social networks [7]. Social trust and social participation, have each been protectively associated with alcohol use among high school students [8].

Binge drinking has a strong social component [9,10]. Adolescents are more likely to drink in social settings, allowing for their drinking habits to be visible to peers. The combination of risk taking and the visibility of alcohol use in peer settings may allow adolescents to maintain their social network status and gain popularity [11]. In addition, some studies have shown that binge drinking varies by gender and socioeconomic status, although these associations are not always consistent.

Because both alcohol use and peer influence increase during adolescence, it is critical to consider longitudinal influences of peer groups on the developmental trajectory of adolescent alcohol use [12]. Furthermore, studies that investigated the association between binge drinking and social capital have not attempted to identify differences among the sub-dimensions of the social capital construct [4,13]. The aim of the present longitudinal study was therefore to investigate the association of social capital with longitudinal changes in the frequency of binge drinking among adolescents at public and private high schools in the city of Diamantina, Brazil.

## Materials and methods

### Study design and sample

To investigate an incidence of binge drinking, a survey was carried out involving all adolescents enrolled in the public and private schools of the city of Diamantina/MG, Brazil, with a full 12 years during the data collection months of the study. Data related to school addresses and number of students enrolled in each class was obtained from the State and Municipal Education Departments.

Subsequently, 633 adolescents from all 13 public and private schools in Diamantina / MG were invited to participate in the study, being previously notified by telephone to schedule the researcher's visit. At that time, the objectives of the research were clarified and what activities would be carried out at the school. Also presented were an approval of the Ethics Committee and as authorizations of the State and Municipal Secretariats of Education. After the consent of the management and the teaching staff, classes with schoolchildren enrolled in public and private schools in the urban area of the city of Diamantina and who were 12 full years on the day of the exam; authorized by the parents / guardians and agreed to participate in the research (inclusion criteria) were contacted by the researcher during class time, with the teacher's presence, for awareness raising. Adolescents not authorized by parents or guardians or who did not agree to participate in the study were excluded from the study. The researcher explained the purpose of the research and asked the students to answer the questionnaires, ensuring the confidentiality of the answers, as well as the evaluation of student participation.

In the baseline survey (2013), the sample consisted of 588 students (participation rate: 92.89%). The reasons for dropouts were non-authorization from parents/guardians or adolescents (4.62%; n = 28) and failure to complete the questionnaires (2.9%; n = 17). In 2014, a new data collection procedure was carried out with these adolescents when they were aged 13 years. Again, all 13 public and private schools in Diamantina / MG were invited to participate in the study and were previously notified by telephone to schedule the researcher's visit. They only included adolescents authorized by their parents or guardians and who agreed to participate in the study. Thus, the follow-up study involved a sample of 588 adolescents (100%). To achieved a 100 percent follow-up rate, the researchers responsible for the data collection made calls to the homes of students who were not present on the day previously scheduled, which led the researchers to return to some schools more than once. Furthermore, access was relatively easy because researchers live in the region and had close contact with directors of the schools.

### Measures

The Alcohol Use Disorder Identification Test (AUDIT C), validated for use in Brazil [14]. was employed for the evaluation of alcohol intake. The AUDIT instrument can identify whether an individual exhibits hazardous (or risky) drinking, harmful drinking or alcohol dependence [15]. AUDIT C (the first 3 questions on the AUDIT instrument, which are related to the frequency and amount of alcohol consumed) was used, as this version can be employed as a stand-alone screening measure to detect hazardous drinkers among adolescents [16,17]: a) “How often did you have a drink containing alcohol in the past year?” b) “How many drinks containing alcohol did you have on a typical day when you were drinking?” c) “How often do you have five or more drinks on one occasion?” The latter item was used to identify binge drinking [18]. The response options are never, less than monthly, monthly, weekly and daily or nearly daily. Responses of “never” were coded as 0 in the analysis. “Less than monthly” and “monthly” were coded as 1. “Weekly” and “daily or nearly daily” were coded as 2. Although the AUDIT C was used to measure alcohol involvement, the dependent variable was change in alcohol consumption, calculated from the difference in consumption observed between 2013 and 2014, categorized into “reduced or unaltered frequency intake” and “increased frequency intake” was based only on AUDIT binge item ([c]).

Our predictor variables included sociodemographic and economic characteristics (gender, type of school, mother's education, family income) and Social Capital.

For evaluation of social capital, we used the Social Capital Questionnaire for Adolescent Students (SCQ-AS), which was developed and validated by our research team [19]. The study population included in the development and validation of the instrument was a convenience sample made up of 101 students aged 12 years enrolled in the public and private school systems in city of Diamantina/MG, Brazil. This questionnaire is composed of items selected from the national and international literature and has been submitted to face validation, content analysis and analyses of internal consistency (Cronbach's alpha: 0.71), reliability and reproducibility (Kappa coefficient’s range: 0.63 to 0.97) [19]. The factor analysis grouped the 12 items into four subscales: Social Cohesion at School; Network of Friends at School; Social Cohesion in the Community/Neighborhood; and Trust at School and in the Community/Neighborhood. Social capital scores range from 12 to 36 points, with a higher score denoting higher social capital (Table 1). As a questionnaire designed for children and adolescents, the decision was made to use a three-point Likert scale with response options of *I agree*, *I do not agree or disagree* and *I disagree*. This procedure was based on the target age group and was chosen to avoid confusion during the filling out of the questionnaire. The findings confirm indications in the literature that networks of friends and neighborhood cohesion reflect experiences one shares with one’s peers and underscore the importance of the present questionnaire as an assessment tool for measuring social capital. Based on the distribution, to analyze the social capital by the adolescent the social capital variable was dichotomized by median as high (31 points or more) and low (less than 31 points). The difference of the social capital at the follow-up in relation to the social capital at the baseline of each adolescent was calculated to obtain the difference between the measures of social capital in the two evaluations. Thus: total score of social capital at follow-up (FSC) minus total score of social capital at the baseline (BSC) presented three response options *increase* in social capital (FSC> BSC), *reduction* (FSC <BSC) and *unaltered* (FSC = BSC). We treated sex, type of school (public or private), maternal education and family income as time invariant.

**Table 1:** Social capital questionnaire for adolescent students and it’s four subscales.

Social Capital Questionnaire for Adolescent Students (total score: 12 to 36)
**School cohesion (score: 4 to 12)**
1. The students at my school stay together
2. I feel like I belong at this school, as if it were mine
3. I feel safe at this school
4. My parents get along with my teachers
**School friendships (score: 3 to 9)**
5. The students at my school have fun together
6. I trust my friends at school
7. I can ask my friends at school for help
**Neighborhood social cohesion (score: 2 to 4)**
8. I trust my neighbors
9. I can count on my neighbors for help
**Trust: school / neighborhood (score: 3 to 9)**
10. The teachers at my school are sympathetic and give us support
11. My neighbors would try to take advantage of me
12. My classmates would try to take advantage of me

### Statistical analysis

Data analysis was performed using the Statistical Package for the Social Sciences (SPSS for Windows, version 22.0, SPSS Inc, Chicago, IL, USA) and included frequency distribution and association tests. The chi-square test was used to determine the statistical significance of associations between binge drinking and the independent variables (p < 0.05). Given the high prevalence of the outcome (> 20%), we used log-binomial model to calculate prevalence ratios (PR) and 95% confidence intervals [20]. In this study, log binomial models were used to calculate both univariate and multivariable models [20]. The two-tailed *p* value was set at <0.05.

### Ethical considerations

This study received approval from the Human Research Ethics Committee of the Federal University of Minas Gerais (Brazil) (COEP-317/11). All parents/guardians signed a statement of informed consent authorizing the participation of their children. All adolescents also signed a statement of assent.

## Results

The sample comprised 588 students (participation rate at one-year follow-up: 100%). Boys accounted for 48.6% (n = 286) of the sample. Among these participants, the vast majority attended public schools (92.2%; n = 542). A total of 75.2% (n = 442) of adolescents were from families that earned up to three times the Brazilian monthly minimum wage, and 61.60% (n = 361) of the mothers had less than eight years of schooling (Table 2).

**Table 2:** Distribution of adolescents according to demographic, socioeconomic characteristics and prevalence of binge drinking (Lifetime), 2013 (Diamantina, Brazil).

Independent variables			Binge drinking	
		N(%)	Yes(%)	No(%)
Gender	Male	286(48.6)	79(27.6)	207(72.4)*
	Female	302(51.4)	57(18.9)	245(81.1)
Type of school	Public	542(92.2)	133(24.5)	409(75.5)*
	Private	46(7.8)	3(6.5)	49(93.5)
Mother’s education (years)	0–7	361(61.60)	92(25.48)	269(74.52)
	8 or more	225(38.40)	49(19.11)	182(80.89)
	missing	2(0.3)		
Family income (wages) ^+^	½-3	442(75.2)	106(24.0)	336(76.0)
	More than 3	145(24.7)	30(20.7)	115(79.3)
	missing	1(0.1)		
Social capital	High	415	77(18.6)	338(81.4)*
	Low	167	55(32.9)	112(67.1)

*Chi-square test

^+^ Minimum Wages in Brazil in 2013: 678 BRL/Month ~ 330,73 USD/Month

The prevalence of binge drinking in 2013 was 23.1% and in 2014 the prevalence had risen to 30.1%, i.e. there was a 7% increase in the prevalence of binge consumption in the period. Of the 452 teens who reported never consuming five or more alcoholic drinks at one time in 2013, 41 started to do so with some frequency in 2014 (Table 3).

**Table 3:** Percentage of the sample related to binge drinking that moved from one category to another between baseline (2013) and follow-up (2014), Diamantina, Minas Gerais, Brazil.

Binge drinking at baseline (%)	Binge drinking at follow-up				
	Never	Less than once per month	Once per month	Once per week	Total
Never	403 (89.2)	45 (10.0)	3 (0.7)	1(0.2)	452 (100.0)
Less than once per month	4 (9.8)	34 (82.9)	3 (7.3)	0 (0.0)	41(100.0)
Once per month	3 (4.0)	46 (61.3)	23 (30.7)	3 (4.0)	75 (100.0)
Once per week	1 (5.0)	8 (40.0)	8 (40.0)	3 (15.0)	20 (100.0)
Total	411 (69.9)	133 (22.6)	37 (6.3)	7 (1.2)	588 (100.0)

According with the changes in the score of social capital total between baseline (2013) and follow-up (2014), 340 (58.4%) adolescents unaltered their social capital total in follow-up; 184 (31.6%) students showed an increase in social capital total in follow-up and 58 (10.0%) showed a reduction in follow-up. Six students did not adequately answer the questionnaire.

Table 4 shows the percentage of the sample related to the in subscales of social capital between baseline and follow-up and its association with the difference on binge drinking between baseline and follow-up. 166 (28.3%) students increased their social capital in the ‘Social Cohesion at School’ subscale and 457 (78.0%) reduced or unaltered their score of social capital total between baseline and follow-up in the ‘Network of Friends at School’ subscale. Twenty-six (21.4%) adolescents who reported an increase in the ‘Social Cohesion in the Community’ subscale also showed an increase in binge drinking at the follow-up and 188 (95.4%) reported a reduction in the ‘Trust’ subscale and in the binge drinking at the same time (Table 4).

**Table 4:** Association between the difference in subscales of social capital between baseline and follow-up and the difference on binge drinking between baseline and follow-up, Diamantina, Minas Gerais, Brazil.

Difference in categories of Social Capital between baseline and follow-up		Total sample (%)	Difference of binge drinking between baseline and follow-up		Total
			Reduced or unaltered	Increased	
Social Cohesion at school	Increased	166(28.3)	144(87.0)	22(13.0)	166(100.0)
	Reduced	71(12.1)	67.4(94.3)	4(5.7)	71(100.0)
	Unaltered	351(59.6)	328(93.4)	23(6.6)	351(100.0)
Network of friends at school*	Increased	129(22.0)	110(85.2)	19(14.7)	129(100.0)
	Reduced or unaltered	457(78.0)	427(93.4)	30(6.5)	457(100.0)
Social cohesion in the community	Increased	121(20.6)	95(78.5)	26(21.4)	121(100.0)
	Reduced or unaltered	467(79.4)	444(95.0)	23(5.0)	467(100.0)
Trust*	Increased	179(30.5)	168(93.9)	11(6.1)	179(100.0)
	Reduced	197(33.6)	188(95.4)	9(4.6)	127(100.0)
	Unaltered	210(35.4)	181(86.1)	29(13.9)	210(100.0)

* Two students did not adequately answer the questionnaire

Log-binomial model shows the incidence of binge drinking according to the background characteristics of the respondents. Gender (PR 0.67; 95% CI 0.40–1.13) and socioeconomic status (type of school and mother’s education) were not associated with the increase in the frequency of binge drinking. However, social capital was significantly associated with an *increase* in binge drinking by students (Table 5).

**Table 5:** Log-binomial model of the changes in prevalence of binge drinking and independent variables among adolescents of Diamantina, Minas Gerais, Brazil, 2014.

Independent variables		Prevalence Ratio (95%CI)^+^	p-value
**Demographic**			
Gender	Male	0.67(0.40–1.13)	0.138
	Female	1	1
**SES**			
Type of school	Public	0.50(0.24–1.04)	0.066
	Private	1	1
Mother’s education (years)	0–7	0.85(0.48–1.49)	0.579
	8 or more	1	1
**Social Influence**			
Social Capital	Increased	5.67(3.13–10.29)	0.000
	Reduced or unaltered	1	1

^+^ Adjusted for all the variables listed in the table

Table 6 shows the prevalence ratios of changes in the frequency of binge drinking according to social capital subscales. Adolescents who reported that they had an increase in social cohesion in the community/neighborhood subscale were 3.3 times more likely (95%CI 1.83–6.19) to binge drink themselves. In addition, adolescents who reported that they had a decrease in trust subscale were less likely (PR 0.4 95%CI 0.21–0.91) to binge drink themselves. However, social cohesion at school and network of friends at school subscales were not associated with the outcome.

**Table 6:** Social capital subscales related to changes in frequency of binge drinking among adolescents in Diamantina, Minas Gerais, Brazil (n = 588), 2014.

Independent variables		Prevalence Ratio (95%CI)	p-value
**Social Capital**			
Social Cohesion at School	Increased	1.26(0.72–2.21)	0.409
	Reduced	1.27(0.44–3.67)	0.656
	Unaltered	1	1
Network of Friends at School	Increased	1.30(0.74–2.27)	0.353
	Reduced or unaltered	1	1
Social Cohesion at the Community	Increased	3.37(1.83–6.19)	0.027
	Reduced or unaltered	1	1
Trust	Increased	0.60(0.30–1.17)	0.135
	Reduced	0.44(0.21–0.91)	0.027
	Unaltered	1	1

## Discussion

The present study examined the frequency of binge drinking among adolescents at public and private schools in the city of Diamantina (southeastern Brazil). The increase in the frequency of binge drinking in the follow-up period was 7% and this increase was fivefold greater among adolescents who exhibited an increase in social capital. Our social capital questionnaire was designed so that we can distinguish the influence of social capital in different contexts that the adolescent is exposed to, i.e. the school environment versus the neighborhood environment. We therefore analyzed the subscales separately. Our findings suggest that adolescents’ drinking behavior is more responsive to changes in the neighborhood context and trust, rather than the school context and friendship network at school. The literature suggests that the concept of social capital can be broken down into ‘structural’ and ‘cognitive’ social capital [21]. Structural aspects of social capital refer to roles, rules, precedents, behaviours, networks and institutions. These may bond individuals in groups to each other, bridge divides between societal groups or vertically integrate groups with different levels of power and influence in a society, leading to social inclusion. By contrast, cognitive social capital taps perceptions and attitudes, such as trust toward others that produce cooperative behaviour [22].

In contrast to the results of the present study, previous reports found that students from U.S. colleges with higher levels of social capital were at *lower* risk for binge drinking [5,23]. The discrepancy may be due to differences in the aspects of social capital examined in the different settings. Specifically, the study based on binge drinking in US colleges focused on the structural aspect of social capital—as measured by the participation of students in voluntary activities. [23]. However, students in our Brazilian sample were at greater risk of binge drinking if they reported higher social capital in the cognitive dimension, i.e. feelings of more cohesion in their communities and neighborhoods and they are less likely to binge drinking if they have a decrease in the trust subscale. The difference between these studies, including the age of the subjects, underscore the observation that social capital can have both positive and negative health implications, depending on the form it takes [24]. In samples with older adolescents who binge drinking more often, we may find a richer (e.g., expected gender effects) and possibly more intuitive pattern of results. Individuals who have higher levels of social support and community cohesion generally are thought to be healthier because they have better links to basic health information, better access to health services, and greater financial support with medical costs [7]. However, it is important to consider the impact of complex community factors on individual behaviors. Some factors as social stratification (i.e., the probability of living in certain neighborhoods, which is higher for certain types of persons) and social selection (i.e., the probability that drinkers are more likely to move to certain types of neighborhoods) may affect health risk behaviors, including alcohol use [7]. In addition, previous research highlighted the importance of having trust in the peers with whom adolescents drank alcohol [25]. Young usually drink more with peers whom they trust probably because of a tacit acknowledgement that a friend understood unspoken rules and could be relied upon [25].

Past studies have found that binge drinking is usually performed in groups; therefore, peers play an important role in promoting binge drinking, perhaps due to peer selection or peer influence (socialization) [4, 23]. Our results show that social cohesion in the community/ neighborhood subscale was significantly associated with increase in binge drinking and a decrease in trust subscale was related to the decrease in frequency of binge drinking among scholars. Although the literature is well established in relation to peer influence on binge drinking, social cohesion at school and network of friends at school subscales were not associated with the outcome. Drinking is viewed by young people as a predominantly social activity which provides an opportunity for entertainment and bonding with friends [25]. During lifetime, friendships can direct development through support, modeling, and assistance, but the significance of friendships is heightened during adolescence [26]. Previous study showed that adolescents’ baseline alcohol use status (drinker/ nondrinker) strongly predicted acquisition of friends exhibiting similar alcohol use patterns twelve months later [27]. Another study among young students [28] that analyzed individual and contextual risk factors for alcohol use (temperamental disinhibition, authoritarian and authoritative parenting, and parental alcohol use) assessed during childhood and adolescents revealed significant variability in the association between alcohol consumption and deviant friends and that deviant friends was a significant covariate of alcohol consumption. Furthermore, this study revealed a significant interaction of Disinhibition × Parental Alcohol Use; the childhood disinhibition interacted with parental alcohol use to moderate the covariation of drinking and deviant friends [28]. The relationship between social environments and binge drinking is complicated in part because of reverse causality or simultaneity. Environmental factors (i.e. school and neighborhood characteristics) may be spuriously linked to binge drinking because, for example, adolescents who live in neighborhoods where violent crime is high and access to illicit substances is easy, may be less likely to be socially connected and more likely to consume alcohol. [29].

Despite being a well-established determinant, the influence of socioeconomic status on health is not well understood and little research has focused on the effects of this aspect on health during adolescence [30]. In the present study, the socioeconomic status was not associated with the increase in the frequency of binge drinking among adolescents. Some studies have demonstrated that adolescents from higher socioeconomic status (SES) backgrounds have a greater propensity to use alcoholic beverages and to engage in binge drinking [4,31,32]. This may be because of higher discretionary income (pocket money) or easier access to alcohol in their homes. However, other studies have found an association between lower socioeconomic status and greater alcohol consumption [16,33], and still others have found no significant association between socioeconomic status and alcohol intake [34,35]. The literature highlighted that differences in results may be partially explained by the use of different indicators adopted such as family income, social class, level of schooling, school type, as well as the considerable variation in cut-off points, as well as the specific culture and the age of the drinker.

In the present study, we did not find statistically significant difference between incidence of an increase in binge drinking and gender. This may be explained by changing gender norms over time, which has made it more acceptable for girls to engage in risky behaviors [36]. In accordance with our results, a longitudinal study used a national data to describe gender differences in health behavior of adolescents and, found that in the case of binge drinking, girl’s behaviors have converged with the rates among boys [36].

A limitation of our study is that as the data were derived from self-administered questionnaires, lack of attentiveness should be taken into consideration. Second, despite emphasizing the importance of giving honest responses, the findings may have been underestimated due to self-censoring and/or a suspicion that school authorities could gain access to the answers on the questionnaires. Third, information on the influence of friends and characteristics of friendship networks, such as density, size, quality of contacts, proximity and centrality, was not collected in the present study, despite the fact that binge drinking has been associated with such factors [1–4, 12, 13]. The aim of the questionnaire was to measure social capital that was easily understood and applicable to adolescent students that encompasses the different domains of social capital for this population. Even though this questionnaire did not measure characteristics of friendship networks, such as density, size, quality of contacts, proximity and centrality, it is measures contexts that involve social relationships, such as experiences at school and in the local community, which can exert an influence on the behavior and decisions of adolescents, thereby reflecting health determinants. The Social Capital Questionnaire for Adolescent Students (SCQ-AS) demonstrate that this assessment tool is appropriate for epidemiological studies involving samples of adolescents in the investigation of the association between social capital and risk factors or health determinants. Finally, we cannot generalize findings from this study to older adolescents within Brazilian culture.

## Conclusion

Binge drinking involves groups of inter-connected people who evince shared behaviors, and is a public health and clinical problem. Targeting these behaviors should involve addressing groups of people and not just individuals [24]. Our results provide new evidence about the “dark side” of social cohesion in promoting binge drinking among adolescents. Social capital interventions must include school and community engagement, parental involvement, and peer participation components to address the complex array of factors that influence adolescent alcohol use.

## Supporting information

S1 File Binge drinking and social capital data. (XLS)
